# Transferrin Receptor-Targeted Nanocarriers: Overcoming Barriers to Treat Glioblastoma

**DOI:** 10.3390/pharmaceutics14020279

**Published:** 2022-01-25

**Authors:** Maria João Ramalho, Joana Angélica Loureiro, Manuel A. N. Coelho, Maria Carmo Pereira

**Affiliations:** LEPABE—Laboratory for Process Engineering, Environment, Biotechnology and Energy, Faculty of Engineering, University of Porto, Rua Dr. Roberto Frias, 4200-465 Porto, Portugal; mjramalho@fe.up.pt (M.J.R.); joana.loureiro@fe.up.pt (J.A.L.); mcoelho@fe.up.pt (M.A.N.C.)

**Keywords:** brain delivery, blood-brain barrier, functionalized nanoparticles, surface modification, active targeting, transferrin, monoclonal antibody, targeting peptides

## Abstract

Glioblastoma multiforme (GBM) is the most common and lethal type of brain tumor, and the clinically available approaches for its treatment are not curative. Despite the intensive research, biological barriers such as the blood–brain barrier (BBB) and tumor cell membranes are major obstacles to developing novel effective therapies. Nanoparticles (NPs) have been explored as drug delivery systems (DDS) to improve GBM therapeutic strategies. NPs can circumvent many of the biological barriers posed by this devastating disease, enhancing drug accumulation in the target site. This can be achieved by employing strategies to target the transferrin receptor (TfR), which is heavily distributed in BBB and GBM cells. These targeting strategies comprise the modification of NPs’ surface with various molecules, such as transferrin (Tf), antibodies, and targeting peptides. This review provides an overview and discussion on the recent advances concerning the strategies to target the TfR in the treatment of GBM, as their benefits and limitations.

## 1. Introduction

Glioblastoma (GBM) is the most frequent and malignant brain tumor in adults, accounting for up to 50% of the primary brain tumors. GBM has a poor response to the clinically available therapies, therefore having a poor prognosis, frequent recurrence, with a 3–5% survival rate at 5 years, and a median life expectancy of 12–15 months [1]. GBM exhibits unique features that challenge the development of effective therapies, such as the diffuse infiltration into the surrounding healthy tissues, the genomic instability, the high vascularization, the intratumor heterogeneity, and the intrinsic resistance mechanisms. The tumor anatomic location hampers its complete surgical resection, requiring further radiotherapy with coadjuvant chemotherapy cycles. The limitations of available chemotherapeutic drugs, such as the low bioavailability, poor pharmacokinetics, and toxicity to healthy tissues, are other obstacles to the success of the current therapeutic approaches. Furthermore, the blood–brain barrier (BBB) is also a challenge for delivering chemotherapeutic agents to brain tumors. Therefore, the search for alternative treatments to effectively cure GBM is one of the most pressing challenges in the medical and scientific communities [2].

The use of functional nanoparticles (NPs) with brain targeting ability is the most researched strategy to tackle these issues. Drug encapsulation in nanosized carriers can enhance the drug bioavailability through the increased accumulation in tumor cells while decreasing undesired side effects in the healthy tissues. Passive targeting strategies present numerous limitations since these are specificity limited, and the biological fate of the NPs depends mainly on their physicochemical features. Therefore, active targeting strategies based on receptor-mediated endocytosis are well-accepted approaches. Different molecules have been explored for the targeted GBM tumor delivery, such as CD133 [3] and Integrin α6 [4]. However, the transferrin receptor (TfR) is a more particularly relevant target, as it is overexpressed in the GBM tumor cells [5] and also in the BBB [6]. Different molecules can be used as ligands for NPs functionalization to target the TfR, such as transferrin (Tf), antibodies, and targeting peptides [7].

This review presents the targeting strategies explored for drug delivery using NPs for GBM therapy. The role of the TfR as a target and the use of different receptor binding molecules, including Tf, anti-TfR antibodies, or TfR-binding peptides, are here discussed. The systematic literature search was conducted using PubMed, Science Direct, Google Scholar, SCOPUS and Web of Science as online databases until November 2021. Only papers published in Quartil (Q) 1 and Q2 journals and written in English in the last ten years were considered.

## 2. Glioblastoma and Limitations of Current Therapy

Glioblastoma (GBM) tumors are grade IV astrocytomas, and this designation is attributed to being originated from astrocytes. Astrocytes are the most abundant glial cells and confer physical and metabolic support for neurons, performing several vital functions for the normal neuronal development. GBM can be divided into: (i) primary tumor that develops rapidly de novo, without a precursor lesion, corresponding to the majority of the cases; (ii) or secondary GBM that progresses from a low-grade astrocytoma (10% of the cases). Despite being histologically and morphologically similar, these two types of GBM present different biomarkers as different genetic and epigenetic profiles [8].

The Stupp protocol is the gold-standard treatment for GBM and consists of surgical resection followed by the combination of radiotherapy and chemotherapy with temozolomide. However, this therapeutic regimen has several limitations, such as brain tissue damage, deleterious side effects, and drug resistance, failing to cure this devastating disease [9]. The extensive diffusion into adjacent tissues and the increased vascularization verified in tumor tissues are major therapeutic limitations. In addition, the unique features of GBM, such as its anatomic location, contribute to these poor therapeutic outcomes.

Although intensive research is being conducted to develop new therapeutic approaches, most new therapies fail in clinical trials, and there is still no cure for this devastating disease. The BBB is still a major obstacle and is responsible for the insufficient delivery and accumulation of several drugs in the brain tumor, hampering the design of new therapeutic molecules. However, recent insights into tumor characteristics and behavior have paved the way for developing promising new approaches, such as targeted and specific therapies and the use of drug delivery systems (DDS).

### Blood-Brain Barrier

**Figure 1:**
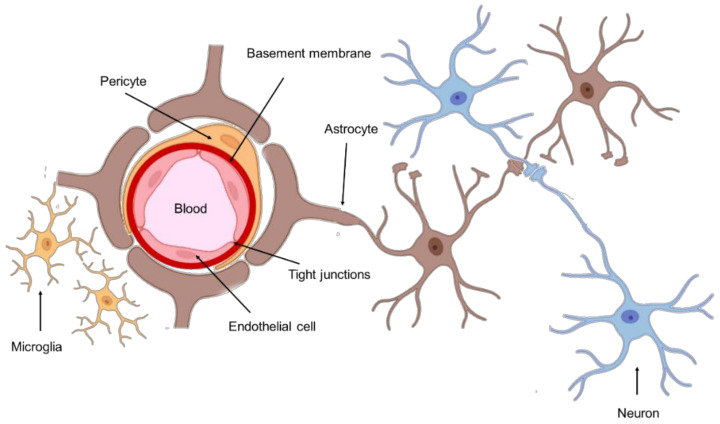
Schematic representation of the BBB.

The anatomic location of GBM tumors enforces the need for therapeutic agents to cross the BBB to be successfully delivered into the tumor site. This barrier comprises the outer lining of blood vessels in the brain and spinal cord, being composed of two membranes—luminal and abluminal—of capillary endothelium, composed of different cell types such as endothelial cells with elaborated tight junctions, pericytes, astrocytes, and microglial cells (Figure 1) [10]. A healthy BBB exhibits low permeability and acts as a shield to the CNS, protecting against drugs and other neurotoxins. Although this structure is vital for homeostasis maintenance and brain nutrition, this poses a significant obstacle in the treatment of GBM.

In GBM, the blood vessels are highly disorganized, affecting the normal vascular organization and function, resulting in a much more permeable structure, the blood-brain tumor barrier (BBTB) [11]. However, due to its high invasiveness, GBM tumor cells proliferate and diffuse beyond the disrupted BBTB, where the function of the BBB is still intact, obstructing the delivery of drugs to the entire tumor extent. In addition, receptors for drug efflux are expressed at the BBTB, thereby ensuring GBM resistance to chemotherapy [12].

The transport of chemotherapeutic drugs through this barrier depends mainly on their physicochemical properties and can occur through two main mechanisms: passive transport or active transport. Passive transport only allows the diffusion of water-soluble compounds and the transcellular transport of small lipophilic molecules (less than 500 Da). Active transport pathways include transcytosis mediated by membrane protein carriers of small molecules and transcytosis mediated by the adsorption of positively charged peptides. However, the receptor-mediated transcytosis of macromolecules is the most relevant mechanism, and the BBB and BBTB cells express a wide variety of receptors in their membrane. Low-density lipoprotein, transferrin, and insulin receptors are the most highly expressed receptors in the BBB cell membrane [13]. Receptor-mediated transcytosis requires the binding of macromolecules to a specific receptor on the cell, inducing endocytosis and subsequent transcytosis. Therefore, strategies to target the membrane receptors of BBB and BBTB cells are being widely explored. These targeting approaches allow one to improve drug biodistribution in the brain tumors by enhancing drug delivery across these barriers and therefore are further discussed in the following sections.

## 3. NPs as Drug Delivery Systems for GBM Therapy

The current therapeutic management of GBM is insufficient, raising the need for new therapies. Nanomedicine has dictated trends for cancer treatment and diagnosis the last decades, and NPs as DDS have been explored as a promising approach for novel GBM therapies. Drug encapsulation in DDSs can improve drug bioavailability in the tumor tissues and circumvent multidrug resistance (MDR), increasing therapeutic efficacy and reducing dose-dependent side effects.

**Figure 2:**
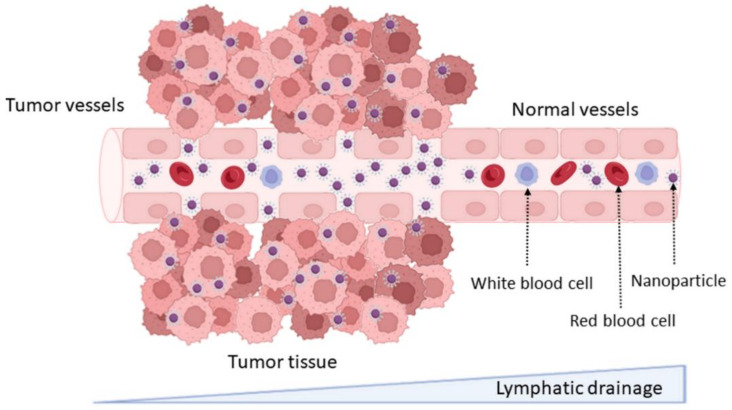
Schematic representation of the enhanced permeability and retention effect and passive targeting mechanisms.

DDS can increase drug accumulation in tumor cells due to the physiological differences between healthy and tumor tissues, including the abnormal and leaky vascularization with increased permeability that is verified in tumors. This effect is known as the enhanced permeability and retention (EPR) effect, and it is as schematized in Figure 2. This passive targeting strategy allows the enhancing of the accumulation of DDS at the tumor tissue [14]. NPs’ accumulation in specific tissues is dependent on their physicochemical properties, mainly their size. NPs under 200 nm were reported to be more easily accumulated in brain tumors, due to the vascular fenestration of the tumor microenvironment (40–200 nm) [15]. The surface charge can also regulate the NPs biodistribution, and neutral and anionic NPs have proven to be more easily transported across the BBB [16].

**Figure 3:**
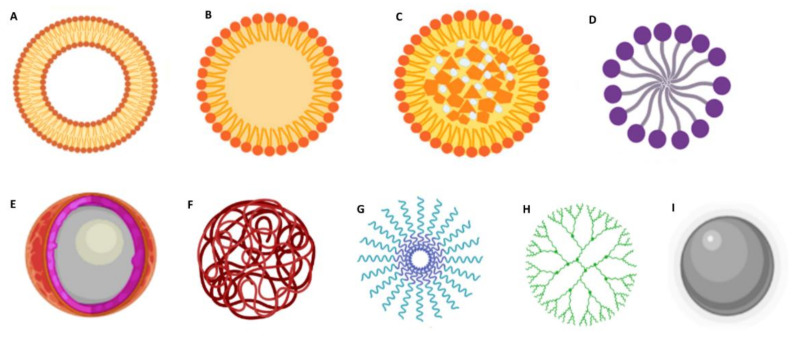
Schematic representation of different types of NPs used in biomedical applications. (**A**) liposome; (**B**) solid lipid NPs; (**C**) nanostructured lipid carriers; (**D**) lipid micelles; (**E**) polymeric nanocapsules; (**F**) polymeric nanospheres; (**G**) polymeric micelles; (**H**) dendrimers; and (**I**) metallic NPs.

Furthermore, DDS should provide structural and chemical protection to the loaded drug and be biocompatible/nontoxic and nonimmunogenic. Other features such as being recognized by the target cells and rapidly internalized by endocytose, exhibiting suitable intracellular trafficking and being biodegradable or easily eliminated, are advantageous [17]. Over the past decades, different types of NPs have been foreseen for GBM therapy. Based on their composition and characteristics, NPs are frequently classified into organic and inorganic [18]. The most studied types of DDS are summarized in Figure 3.

Lipid-based DDS are among the most popular nanocarriers for GBM therapy due to their increased lipophilicity, which confers them the ability to cross the BBB (Figure 3A–D) [19]. Depending on their properties, these can be suitable for encapsulating both hydrophilic and hydrophobic molecules. Liposomes (Figure 3A) are composed of phospholipids that assemble in bilayers concentrically oriented around an aqueous central compartment to protect their nonpolar region from the water [20]. They also have the advantage of being highly biocompatible, biodegradable, stable, and easy to manipulate. Solid lipid NPs (SLNs) are also widely explored for GBM application (Figure 3B). These are composed of a solid hydrophobic lipid core. Depending on the type of lipid, surfactant, and production technique, drugs can be incorporated in the SLNs in different ways: (i) in the homogeneous matrix model, where the drugs are dispersed in the SLN core; (ii) the lipid core is covered by a wall containing the drug; and (iii) the drugs are in the lipid core which is covered by a lipid wall. Nanostructured lipid carriers (NLCs) are composed of a solid lipid matrix and a liquid lipid (Figure 3C). The mixture of the solid lipid with a liquid lipid creates imperfections in the matrix that provides a large space for the entrapment of drug molecules resulting in high drug loading capacities [21]. Lipid micelles (Figure 3D) are spherical amphiphilic assemblies with a hydrophobic core and a hydrophilic shell that provides stability in an aqueous medium. Drugs can be loaded in the central core or covalently linked to the lipids.

Polymeric formulations (Figure 3E–H) can be prepared using a wide range of materials and therefore can be very flexible in composition, structure, and properties [22]. Natural polymers include alginate, chitosan, dextran, hyaluronic acid, and pullulan. The most popular synthetic polymers are poly(lactic-co-glycolic acid) (PLGA) [23], polyethylene glycol (PEG), and poly-ε-caprolactone (PCL) [24]. These polymers can be used to produce polymeric nanocapsules (Figure 3E) that function as a reservoir system where the drug is located in an oil/aqueous core surrounded by a polymeric membrane; nanospheres, where the drug is dispersed within the polymeric matrix (Figure 3F); and polymeric micelles (Figure 3G), where the drug can be entrapped in the hydrophobic core [25]. Dendrimers (Figure 3H) are highly branched polymeric assemblies. Due to their unique features, such as stable structure, small dimensions, monodispersity, aqueous solubility, and high drug loading capacity, these are very popular for nanomedicine applications. However, their extremely small sizes pose a disadvantage, since they are rapidly cleared from blood circulation [18].

Inorganic NPs have also emerged as promising tools, with them being very explored for theranostic applications. These exhibit several interesting physicochemical features, such as optical absorption, fluorescence, and magnetic moment. Metallic NPs (Figure 3I) such as gold NPs and iron oxide NPs are among the most popular inorganic NPs [26,27] and are frequently used as imaging systems for magnetic resonance imaging (MRI), and nanosystems for photothermal therapy [27].

One of the main advantages of organic DDSs is that they typically display terminal surface groups, making them perfect candidates for surface functionalization to increase blood circulation half-life and improve biodistribution and accumulation in tumor tissues. Passive targeting mechanisms are specificity limited, and as the result of the overall physicochemical NPs features, these present numerous limitations. Therefore, active targeting strategies are widely studied to improve the accumulation of NPs at the target site.

Different strategies are being explored for the targeted brain delivery of drugs, such as induced thigh junction opening and nose-to-brain delivery. NPs can be produced (or have their surface modified) with materials with mucoadhesive properties for nose-to-brain delivery, bypassing the BBB and delivering the drug into the brain via the olfactory route [28]. The temporary opening of the BBB tight junctions enables the NPs to penetrate between the endothelial cells [29]. Several modulators can be used to induce this transient opening, ranging from biological elements as viruses and chemicals as cell-penetrating peptides (CPP) to physical stimuli such as focused ultrasound, high frequency, and electromagnetic fields [30].

Surface modification strategies can also be employed for receptor-, adsorptive-, and transporter-mediated endocytosis. Positively charged NPs can cross the BBB by adsorptive-mediated endocytosis due to their electrostatic interactions with the negatively charged BBB [31]. Transporter-mediated endocytosis can be achieved by conjugating the NPs’ surface with molecules that have a transporter highly expressed on the BBB, such as glutathione and choline [32]. NPs can also be modified with CPPs that are positively charged and can penetrate through the cell membranes [33].

Although surface modification for receptor-mediated endocytosis of the NPs is still the most popular strategy for brain tumor-targeted delivery [34], these strategies are further discussed in the following sections.

## 4. Surface Modification Strategies for GBM Tumors Active Targeting

Drug delivery to GBM requires the DDS to be able to cross the BBB and the tumor cellular membrane and to release the drug cargo inside the cell. The passive targeting strategies are insufficient for effective drug delivery, reinforcing the need for active strategies. These are crucial to enhance NPs transport across the BBB and GBM cell membranes. The high surface-to-volume ratio of NPs makes it relatively simple to conjugate ligands. Therefore, ligands that bind specifically to BBB cell receptors [35] and GBM tumor biomarkers have been widely explored for targeted delivery [36].

**Figure 4:**
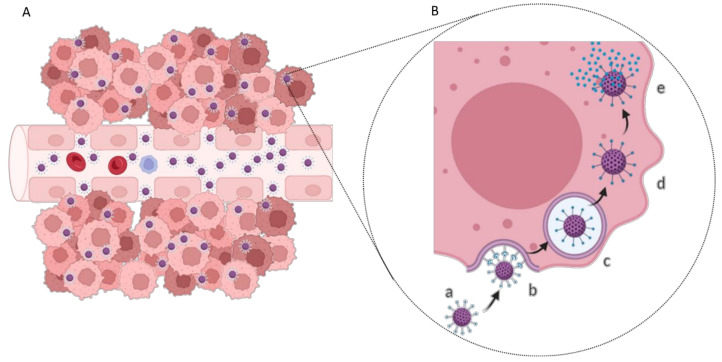
(**A**) Active targeting drug delivery in tumor tissue and (**B**) schematic representation of the receptor-mediated endocytosis process: (**a**) NP modified with ligand; (**b**) NP recognition and binding to the cell membrane receptor; (**c**) NP entrapped in the endosome; (**d**) endosomal escape; and (**e**) drug release into the cell cytoplasm.

GBM cells express specific biomarkers on their membranes in response to the tumor microenvironment or overexpress conventional cell receptors due to abnormal metabolism and proliferation. This facilitates the development of active targeting strategies based on cell uptake by receptor-mediated endocytosis (Figure 4) [37]. The most widely studied targets for GBM therapy are summarized in Table 1.

Different ligands are also explored to target the BBB, with a particular focus on insulin, leptin, insulin-like growth factor, transferrin (Tf), and low-density lipoprotein (LDL) [50]. Cell receptors that are overexpressed in both BBB and GBM cells have been receiving more attention as these can be envisaged for dual-targeting strategies, as is the case of the TfR. TfR is known as one of the major proteins expressed on the luminal side of BBB [51]. Because these receptors are responsible for transferring iron into growing cells, GBM cells overexpress the TfR to meet the increased demand for iron to sustain the rapid cell division [5]. TfR expression on GBM cells is reported to be up to 100-fold higher compared to healthy cells, such as normal human astrocytes [52]. As a result of the differences in the TfR distribution in normal and cancer cells, specific TfR-target strategies can be developed, not only for delivering therapeutic molecules to the brain tumor but also for serving as cancer biomarkers to correlate with tumor staging or cancer progression [51].

Different TfR-targeting strategies have shown promising in vitro and in vivo results, such as increased BBB penetration, cellular accumulation, improved drug delivery to the diseased tissue, and prolonged survival in mice. The TfR targeting may be achieved by using different strategies such as functionalization with Tf itself or monoclonal antibodies or single-chain variable antibody fragments (scFv) to the TfR [53]. The works reported in the last decade for the different strategies to target GBM cells based on the TfR are reviewed in the subsequent sections.

### 4.1. Surface Modification with Transferrin Molecules

Human Tf is a 76-kDa blood–plasma glycoprotein mainly produced in the liver and plays a central role in iron metabolism. Tf is responsible for ferric-ion delivery and plasma Tf can be found in the non-iron bound (apo-Tf), monoferric, or diferric (holo-Tf) forms [54]. Tf is probably the most explored targeting ligand for TfR-mediated brain delivery due to being nonimmunogenic and easily obtained from human sources at a relatively low cost [55]. Therefore, many authors reported the use of this ligand for targeted drug delivery in many applications, such as for GBM therapy. Information regarding some of these studies is compiled in Table 2.

Tf-modified liposomes have been widely studied for brain delivery and are a promising tool for GBM therapy. Jhaveri et al. (2018) developed anionic liposomes to deliver the natural compound resveratrol [59]. The cell internalization and cytotoxicity potential of the Tf-tailored liposomes was evaluated on human GBM cells comparatively with control liposomes (nonmodified). The obtained results proved that surface modification significantly improved cell uptake and the antiproliferative effect. The authors further assessed if the ligand density impacted the targeting ability of the NPs and observed that increasing ligand density enhanced cell uptake up to a ligand intensity of 4 1.5 mol%, after which it caused the reverse effect due to the saturation of the TfR. In vivo studies using mice bearing subcutaneous tumor xenografts depicted that surface modification with Tf improved the tumor growth inhibition and the mice’s survival.

Porru et al. (2014) developed cationic liposomes containing zoledronic acid, and their in vitro cytotoxicity was compared with unmodified liposomes using human GBM cells [57]. The results showed that drug encapsulation in both Tf-modified and unmodified liposomes enhanced its antiproliferative effects. The in vivo therapeutic evaluation of both Tf-modified and unmodified liposomes was conducted using two tumor xenograft models. Intramuscular or intracranial xenografts were established in immunocompromised mice, and the real-time biodistribution of liposomes was evaluated using a noninvasive in vivo imaging system. The obtained results showed that the surface modification led to a higher intratumor localization of NPs both in intramuscular and intracranial xenografts. In addition, Tf-liposomes proved to be more efficient in inhibiting tumor growth and in increasing mice survival in both intramuscular and intracranial xenografts comparatively with unmodified liposomes.

Lv et al. (2013) developed cationic liposomes for the delivery of the anticancer agent cisplatin [56]. In vitro cytotoxicity studies using GBM rat cells (C6) showed that the encapsulation of cisplatin in Tf-modified liposomes increased drug’s inhibitory effect by about 4 times. Using an in vitro BBB model composed of mouse brain microvascular cells (bend3 cells), the authors showed that surface functionalization increased the transport of the liposomes across the BBB model by almost 200%. The authors also verified that Tf-liposomes remained intact after BBB permeation. The authors also established a coculture model (bend3 and C6 cells) to study the ability of the liposomes to sequentially cross the BBB and target the GBM cells. The results showed that Tf-modification enhanced the transport across the BBB and sequential targeting of the C6 cells when compared with unmodified liposomes, leading to increased antiproliferative activity.

Other authors also developed Tf-modified liposomes to encapsulate metallic NPs for dual imaging and treatment agent for glioma. Seleci et al. (2021) entrapped magnetic NPs and quantum dots inside Tf-liposomes [58]. The authors demonstrated that conjugation with Tf increased almost three-fold the nanocarrier internalization, leading to higher antiproliferative activity in human GBM cells. The authors also verified that an external magnetic field significantly increased cell uptake and consequently the antiproliferative activity due to an effect of magnetic guidance, clearly demonstrating the efficient dual-targeting modality of the developed system.

Sonali et al. (2016) also developed Tf-tailored liposomes for theragnostic applications [60]. The group used neutral liposomes for the coencapsulation of quantum dots and the anticancer drug, docetaxel. Biodistribution studies using rats proved that surface modification significantly increased drug accumulation in the brain tissue after i.v. administration, revealing the brain targeting ability of the developed system. The authors did not study the biological performance of the developed nanosystems in GBM models.

Polymeric NPs have also been widely studied for GBM therapy. Liu et al. (2013) developed polylactic acid (PLA) NPs modified with Tf for the delivery of the anticancer drug doxorubicin [61]. In vitro studies using rat glioma cells confirmed the advantages of the surface modification with Tf, with the tailored NPs showing enhanced cell uptake by two-fold and higher antiproliferative activity than the unmodified NPs. Pretreatment with excess Tf to block the cell receptors led to a markedly decreased cytotoxicity of modified NPs. Intracranial tumor-bearing rats treated with NPs by i.v. administration showed higher accumulation of modified NPs in the brain tissue and higher tumor growth inhibition, resulting in increased survival of the treated animals.

Ren et al. (2010) developed unloaded PLA NPs functionalized with Tf [62]. This work aimed to demonstrate the targeting ability of the developed nanocarriers as a proof of concept. In vitro studies using rat glioma cells showed a higher uptake for Tf-coated NPs comparison to PEG-coated and uncoated PLA NPs. Tf-PLA NPs also showed higher tumor-targeting ability than control NPs in rats bearing intracranial tumors without accumulating in the healthy brain tissue.

Other polymers have been proposed to design Tf-coated NPs, such as chitosan, which was proposed by Agrawal et al. (2017) for docetaxel delivery [63]. In vitro cell studies using rat glioma cells showed that drug encapsulation decreases the drug efflux from the cells. The surface modification with Tf was able to significantly increase the NPs’ uptake compared to the nonmodified NPs. Further in vitro cytotoxicity studies revealed that modified NPs were 5 times more efficient than control NPs in inhibiting cell proliferation. The authors also confirmed that modified NPs improved drug pharmacokinetics by increasing blood circulation time after i.v. injection.

Sun et al. (2017) designed PAMAM dendrimers for the Tf-targeted delivery of the gold-standard drug for GBM treatment, temozolomide [68]. Cell uptake and cytotoxicity were evaluated in vitro using patient-derived cells obtained by surgically resected tumor tissue. The authors established two in vitro models from the patients’ samples. In the first, surgical samples were used directly, and in the other, the tumor samples were dissociated for primary neurospheres culture. Despite no significant differences being observed in the cytotoxicity of Tf-modified and nonmodified dendrimer in the treated surgical samples, Tf-surface modification proved to enhance the dendrimers uptake and toxicity in neurospheres culture. In vivo studies revealed the ability of the modified dendrimers to reach the brain tumor tissue after i.v. administration in intracranial tumor-bearing mice, allowing for the specific drug delivery, which led to tumor regression and delayed tumor recurrence, increasing survival rate.

Different groups proposed silicon NPs for Tf-mediated GBM targeting. Sheykhzadeh et al. (2020) developed silicon NPs and evaluated their ability to inhibit cell migration [65]. The authors developed two types of NPs, one functionalized with Tf and control NPs modified with bovine serum albumin (BSA). Surface modification with Tf enhanced by three-fold the cellular uptake by human GBM cells. Cytotoxicity studies showed that the developed NPs were biocompatible. The authors also established an in vitro migration chip model to study cell migration in a confined space representing tumor cell infiltration in brain parenchyma. The obtained results showed that Tf-NPs inhibited cell migration by 40% as compared to control BSA NPs. Furthermore, the authors verified that Tf alone did not inhibit cell migration, suggesting that the inhibitory effect was due to NPs uptake. This work demonstrated the potential of these NPs for clinical application, which can be further strengthened by combining their inhibitory effect on cell migration with their drug-delivery ability.

Luo et al. (2019) proposed silicon NPs modified with Tf as drug delivery systems for doxorubicin [66]. The authors verified that Tf-modification increased NPs permeability across a brain microvascular endothelial cell monolayer used as in vitro BBB model. The developed NPs also exhibited high selectivity to human GBM cells, with surface functionalization increasing cell uptake and cytotoxicity.

Metallic NPs have also been proposed for targeted delivery for GBM therapy, such as iron oxide NPs which have been proposed by Liu et al. (2018) for the delivery of small interference RNA against polo-like kinase I (PLK1) [71]. Silencing the PLK1 gene is an attractive strategy for GBM therapy since it is closely related to tumor progression and recurrence. The authors evaluated the effect of the developed NPs in human GBM cells. The delivery of siRNA by the Tf-modified iron oxide NPs enhanced the gene silencing ability due to a higher transfection activity when compared with control transfection agent-siRNA complex. Furthermore, the authors verified that Tf-tailored NPs exhibited higher antiproliferative activity than unmodified NPs due to the observed increased cell uptake. Tf-coated NPs also showed a higher ability to permeate across the in vitro BBB model than control NPs. Additionally, in vitro studies with 3D spheroid models showed that Tf-modified NPs had higher penetration ability in the spheroid model than unmodified NPs, penetrating a deeper region of the tumor spheroid and resulting in higher antiproliferative activity. Biodistribution studies in mice bearing intracranial tumors showed that the functionalization of the NPs with Tf enhanced the accumulation of the NPs in the brain tumor tissue of the animals, leading to an improved antiglioma efficacy with increased survival of the treated animals.

Zhu et al. (2018) developed ruthenium NPs containing the antitumor drug [Ru(bpy)_2_(tip)]^2+^ (RBT) for photodynamic therapy of GBM [70]. Under laser irradiation, RBT induces the production of reactive oxygen species, inducing cell apoptosis. The developed Tf-modified NPs exhibited a high ability to permeate across an in vitro BBB model composed of human brain microvascular endothelial cells. Furthermore, the authors verified that pretreatment of the BBB monolayer with excess Tf significantly decreased the permeability of the Tf-NPs. In vitro studies with 3D human GBM spheroid models showed that the developed NPs can penetrate the center of the tumor and enhance the antiproliferative activity of RBT by inducing an increase in the intracellular ROS levels. Biodistribution studies after i.v. injection of Tf-NPs in intracranial tumor-bearing mice showed the ability of the NPs to accumulate in the brain tumor, inhibit tumor growth, and increase animal survival.

Other groups developed different Tf-modified NPs for GBM therapy, such as self-assembled near-infrared (NIR) dye NPs composed of indocyanine green. Zhu et al. (2017) proposed these NPs for theragnostic applications [67]. An in vitro cytotoxicity study using in vitro tumor spheroids revealed the biocompatibility and the tumor-penetrating ability of the developed NPs. Furthermore, in vitro uptake studies using GBM cells and mouse brain microvascular endothelial cell monolayers revealed a higher uptake for NPs than free dye. This effect was more pronounced in the GBM cells than in the healthy brain cells, due to the higher expression level of TfR verified in GBM cells, demonstrating the targeting selectivity of the developed NPs. The authors also proved that Tf-NPs enhanced the in vitro photothermal effect of the dye under NIR laser irradiation. Two tumor mice models were established to assess the targeting ability of the developed NPs in vivo, with the dual-modal imaging being evaluated in mice bearing subcutaneous and intracranial tumors. Real-time imaging of subcutaneous tumors revealed that after i.v. injection, NPs accumulated in the tumor tissue 38-fold more than the free IGCG that was detected throughout the whole body, mainly accumulating in the liver and kidney. In the intracranial model, no dye signal was detected in the tumor tissue of mice treated with free dye, reinforcing the need to use NPs to cross the BBB and deliver the dye to the brain tissue. In vivo photothermal therapy experiments showed that the developed NPs can efficiently inhibit tumor growth under laser treatment by converting laser energy into hyperthermia for tumor ablation, increasing animal survival rate while being safe for healthy organs.

**Figure 5:**
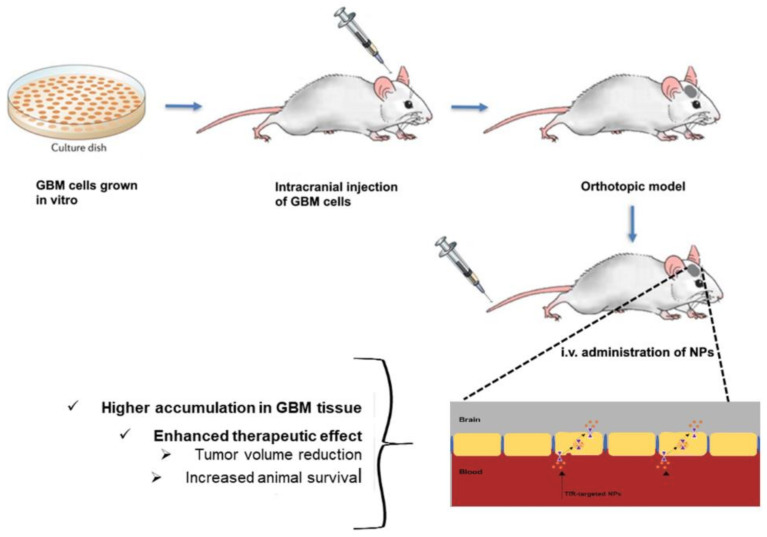
Schematic representation of intracranial tumor mice establishment for TfR-targeted NPs evaluation.

In most of the reported works, the authors established in vivo orthotopic tumor mice models and verified that modification of NPs’ surface with Tf molecules enhanced the GBM targeting ability, leading to better therapeutic outcomes (Figure 5).

### 4.2. Surface Modification with Antibodies against the TfR

NPs can also be tailored with antibodies to target TfR. The use of antibodies, particularly monoclonal antibodies, presents some advantages due to their high specificity, allowing the use of small amounts and still achieving high levels of targeting [72]. Monoclonal antibodies exhibit advantageous features over polyclonal antibodies, exhibiting increased specificity, long half-life, homogeneous structure, and their ability to be mass-produced [73].

Thus, monoclonal antibodies against TfR have been extensively explored for targeted drug delivery in many applications, and different clones have been reported [74,75,76,77], with particular attention for OX26 and RVS10 clones for GBM therapy. In Table 3, the reported works using antibodies for surface modification for GBM therapy are presented.

OX26 antibody has attracted attention from the scientific community. Recently, Ashrafzadeh et al. (2020) developed OX26 modified liposomes for the encapsulation of cisplatin [78]. In vitro cell studies showed that the tailored liposomes were more efficiently internalized by the GBM cells. Biodistribution studies in intracranial tumor-bearing Wistar rats revealed that surface modification with the antibody increased the accumulation of the drug in the tumor tissue in comparison with unmodified liposomes. The higher tumor-targeting ability of the tailored nanocarriers increased the drugs’ efficacy in inhibiting tumor growth and increasing animal survival.

Ramalho et al. (2018) also used the OX26 clone for GBM cells targeting [81]. The group developed temozolomide-loaded PLGA NPs, and in vitro cell studies using human GBM cells depicted that surface tailoring increased the cell uptake of the NPs. The developed NPs showed no toxicity in in vitro studies using a BBB model, proving that the NPs are biocompatible. However, in vitro antitumor experiments revealed that surface modification decreased antitumor efficacy compared to unmodified PLGA NPs, due to the antibody molecules at the NPs’ surface slowing the release of the drug. Further animal experiments would allow the validating of the potential of the developed NPs.

A different clone against TfR, RVS10, was explored by Patil et al. (2010), which modified the surface of poly(β-l-malic acid) NPs containing temozolomide [82]. The developed NPs were efficiently internalized by human GBM cells and enhanced drug activity in vitro.

In recent years, single-chain variable fragments (scFv) against the TfR have been explored in replacement of antibodies. ScFv are produced by fusing the heavy and light chains of antibodies through a short polypeptide linker. These have several advantages over conventional antibodies due to their smaller size, allowing NPs to penetrate tumor tissue more efficiently. Kim et al. (2015) modified the surface of temozolomide-loaded liposomes with a scFv against the TfR [79]. In vitro studies proved that drug encapsulation in tailored liposomes increased tumor cell death due to an enhanced cell targeting ability. In vivo studies using athymic mice bearing intracranial tumors showed that the liposomes could accumulate in the tumor tissue, potentiating the antitumor activity of the drug, leading to an increase in the mean survival of mice. Later, the same group employed these scFv-modified liposomes to deliver the p53 tumor-suppressor gene [80]. Cytotoxicity studies using two human GBM lines previously treated with free TMZ showed that gene transfection with modified liposomes was able to enhance the tumor inhibition activity of the drug. An athymic mouse model bearing intracranial tumor was used for the in vivo evaluation of the developed liposomes. Systemically administered liposomes proved to specifically target the intracranial tumors. Concomitant treatment with temozolomide inhibited tumor growth and prolonged animal survival. These liposomes were the only TfR-targeting nanoformulation for GBM application that reached the clinical trials stage, further discussed in Section 5.

Based on the presented results, the use of antibodies or antibodies fragments to tailor the NPs’ surface proved to be a good approach to deliver anticancer drugs into brain tumors with therapeutic potential.

### 4.3. Surface Modification with Peptides Targeting the TfR

Although targeting cell membrane receptors has been envisaged mostly by using their natural ligands and antibodies or their fragments, in the last years, short peptides have increasingly attracted interest as targeting moieties. These peptides have the particularity of binding to an alternative site of the TfR and not competing with endogenous Tf molecules. These also exhibit higher cost effectiveness comparatively with antibodies [83]. In Table 4, the reported works using TfR-targeting peptides to modify the NPs’ surface for GBM therapy are presented.

Different peptides have been reported for TfR targeting, but T7 (HAIYPRH) and T12 (THRPPMWSPVWP) peptides are undoubtedly the most explored so far. Both peptides were first synthesized by Lee and colleagues (2001) [98] and patented in 2004 (US6743893B2). These synthetic peptides bind to a small cavity on the TfR surface, different from the binding site of Tf. Therefore, these are taken up through receptor-mediated endocytosis while not competing with the endogenous protein [99].

Sun et al. (2020) modified the surface of PLA micelles with the T12 peptide for GBM therapy with paclitaxel [91]. T12 peptide proved to significantly increase the micelles in vitro uptake by human GBM cells compared to unmodified micelles. T12-micelles also exhibited two-fold higher antiproliferative activity than control micelles, by promoting cell apoptosis and inhibiting cell migration in vitro. In vitro studies also revealed that surface modification of the nanocarriers with the TfR-targeting peptide increased transcytosis across a BBB model. In vivo biodistribution studies revealed that T12-micelles have a higher ability to accumulate in the brain tissue than unmodified micelles. Further, modified T12 micelles improved the antitumor activity of paclitaxel with no side effects in mice bearing subcutaneous tumors.

Mu et al. (2017) developed liposomes conjugated with T12 peptide to deliver an alkaloid with anticancer properties, vinblastine [85]. In vitro studies showed that the T12-liposomes could permeate the BBB model and be internalized by glioma cells. Drug encapsulation on the modified liposomes enhanced drug cytotoxicity in human GBM cells. Surface modification of the liposomes enhanced the brain targeting ability as demonstrated in biodistribution studies using mice with intracranial tumors, leading to an enhanced therapeutic effect and increased animal survival rates.

Youn et al. (2014) also modified the surface of NPs with T12 peptide [87]. The group developed nanocomplexes of myristic acid for the delivery of siRNA. Compared with nonmodified nanocarriers, modified NPs exhibited enhanced cellular uptake in human GBM cells and in an in vitro BBB model. In vitro transfection studies also revealed a significant gene silencing activity with T12-nanocomplexes.

Different groups have developed T7-modified PLGA NPs for further GBM therapy application. Cui et al. (2016) employed these NPs to coencapsulate paclitaxel, curcumin, and iron oxide NPs [89]. In vitro cell studies using a human GBM cell line showed that surface modification with the peptide significantly increased the NPs’ uptake compared to the nonmodified NPs. The authors also confirmed that modified NPs have higher brain targeting ability in an in vitro BBB model and in vivo using mice bearing intracranial tumors. Higher NP accumulation in the brain tumor tissue led to an enhanced treatment efficiency with higher survival rates and reduced side effects.

He et al. (2021) used T7-modified PLGA NPs to encapsulate a kinase inhibitor, seliciclib [88]. The therapeutic potential of the developed NP was evaluated in vitro using human GBM cells, and the obtained results showed an improvement of NPs uptake compared with unmodified control NPs due to a high expression of TfR in the used cells (U87). Cytotoxicity studies revealed that drug encapsulation in T7-modified PLGA NPs increased anticancer activity due to the more efficient delivery of seliciclib.

T7 peptide was also used to target other types of polymeric nanostructures to brain tumors, namely poly-l-lysine dendrimers. In fact, Liu et al. (2012) developed cationic dendrimers for the codelivery of doxorubicin and the pORF-hTRAIL gene [93]. The authors aimed to achieve a synergistic effect to enhance the antitumor effect via the tumor necrosis factor (TNF)-related apoptosis-inducing ligand (TRAIL), which is encoded by the encapsulated gene. The surface modification of the developed dendrimers with T7 peptide improved the cellular uptake and gene expression compared with unmodified dendrimers. Biodistribution studies using mice bearing intracranial tumors showed the higher ability of the T7-modified dendrimers to accumulate in the brain tissue, reducing the systemic toxicity in healthy tissues. Tailored dendrimers prove to increase the therapeutic efficacy by a synergistic effect and increase the animal survival time by two-fold, compared with unmodified dendrimers.

**Figure 6:**
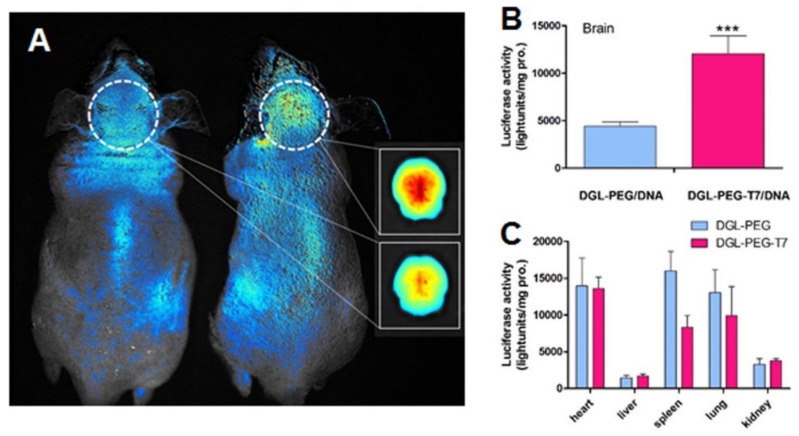
(**A**) In vivo imaging after i.v. administration of ethidium monoazide bromide-labeled control dendrimers (left) and T7-modified dendrimers in nude mice. The inset show the relative brain accumulation of control (upper) and T7-modified dendrimers (lower). (**B**,**C**) The quantitative evaluation of gene expression in vivo. Luciferase expression 48 h after i.v. administration of control and T7-modified dendrimers into nude mice. Luciferase expression of brain (**B**) and other solid organs (**C**) is plotted as light units per mg protein. *** *p* < 0.001 compared with the control dendrimers. Data are expressed as mean ± SD (*n* = 4). Reproduced with permission from [94], published by Elsevier, 2013.

Kuang et al. (2013) also developed T7 peptide-modified poly-l-lysine dendrimers for GBM therapy [94]. The authors proposed this system for the delivery of siRNA, and in vitro studies using a human GBM cell line depicted that modified dendrimers were more efficiently internalized by the target cells than unmodified dendrimers. In vitro studies also revealed that the T7-dendrimers are internalized by receptor-mediated endocytosis and can successfully escape from the lysosomes into the cytoplasm of the cells. In vivo studies showed that surface modification with the peptide increased the dendrimers accumulation in the brain of mice with intracranial tumors by almost three-fold as shown in Figure 6A. This improved GBM-target ability enhanced the gene silencing activity of the tailored nanocarriers in the brain when compared with control dendrimers (Figure 6B).

T7 peptide was also employed to modify lipid nanocarriers for GBM therapy. Wei et al. (2016) loaded siRNA into cationic liposomes aiming to downregulate the expression of the epidermal growth factor receptor (EGFR) that is upregulated in GBM tumors and is involved with its pathogenesis [84]. T7-tailored liposomes showed a higher ability to permeate across the in vitro BBB model and to be internalized by the human GBM cells. The developed liposomes enhanced the gene silencing activity of the entrapped siRNA in vitro and in vivo. In vitro studies with 3D spheroid models showed that T7-modified liposomes have higher penetration ability than unmodified liposomes, penetrating a deeper region of the tumor spheroid. In vivo biodistribution studies showed a higher accumulation of modified liposomes in the brain tissue of mice bearing intracranial tumors, leading to an enhanced antiglioma efficacy with increased survival of the treated animals and lower toxicity in healthy organs.

Fu et al. (2019) developed anionic SLNs modified with T7 peptide to deliver the anticancer alkaloid, vincristine [86]. The developed SLNs were coated with red blood cell membranes (RBCs) to enhance circulation half-life due to their lower immunogenicity compared to synthetic materials. RBCs display several advantages for NPs’ coating, allowing for the NPs to maintain their physicochemical properties required for efficient drug delivery while providing biological functions of natural cell membranes. However, RBCs exhibit insufficient targeting specificity, and thus, modification with a TfR-targeting peptide is still needed. A dual-targeting strategy was employed in this work, and the SLNs were also modified with Asn-Gly-Arg (NGR). This negatively charged peptide is a ligand of CD13, a transmembrane metalloprotease, that is overexpressed in GBM tumors. The developed dually tailored SLNs (T7 + RGD-SLNs) and T7-SLNs exhibited high permeation across an in vitro BBB model, contrary to what was verified in NGR-modified SLNs, proving the brain targeting ability of T7 peptide. The authors also demonstrated that the T7 + RGD-SLNs were able to increase the cellular toxicity of vincristine in rat glioma cells by improving cell uptake in the tumor cells. The authors further observed that increasing ligand density enhanced cell uptake up to a ligand intensity of 4% molar proportion, after which it caused the reverse effect due to the saturation of the TfR. Biodistribution studies in mice bearing intracranial tumors showed that while unmodified and NGR-modified SLNs possessed a reduced brain-targeting ability, T7-functionalized and T7 + NGR-SLNs were accumulated in a higher extension in the brain of mice. This higher brain targeting ability of the dually tailored SLNs resulted in higher tumor growth inhibition and increased animal survival.

T7 peptide was also employed for the surface modification of metallic NPs, such as gold NPs as proposed by Dixit et al. (2015) for the delivery of a photosensitizer phthalocyanine 4 (Pc 4) [96]. In vitro studies using human GBM cells, showed that tailoring the NPs with T7 peptide doubled the cell uptake, suggesting that the NPs are internalized by Tf receptor-mediated endocytosis. Cytotoxicity studies conducted in the presence and absence of light showed that the NPs per se do not produce toxicity, being biocompatible, but surface modification enhances the light-induced cytotoxicity of Pc 4. Biodistribution studies using intracranial tumor-bearing mice proved that surface modification significantly increased by six-fold the drug accumulation in the brain tumor tissue after i.v. administration.

This peptide has also been used for the functionalization of naturally occurring DDS. Exosomes are naturally occurring nanosized vesicles composed of natural lipid bilayers and proteins that readily interact with cell membranes and have been recently explored for drug delivery [100]. Kim et al. (2020) designed exosomes containing antisense microRNA oligonucleotides and decorated them with the T7-peptide [97]. The authors aimed to selectively deliver antisense oligonucleotide against miR-21 that is commonly upregulated in GBM and involved in the inhibition of tumor cell death and consequent tumor progression. T7-modified exosomes exhibited higher cell uptake in rat GBM cells than unmodified exosomes. Further in vivo studies using rats with intracranial tumors showed that T7-exosomes are more efficiently accumulated in the brain tissue than control exosomes, leading to a higher reduction in the miR-21 levels. Exosomes have attracted significant attention as drug delivery due to their ability to avoid recognition by the host immune system and enhance delivery of entrapped drugs to target cells.

The conjugation of nanosystems with TfR-targeting peptides proved to be a suitable strategic approach to improve the therapeutic efficacy of anticancer drugs for treating brain tumors while decreasing toxicity for healthy organs. However, l-formed peptides were used in all of these works. As l-peptides are often susceptible to degradation by proteolytic enzymes, a retroinverse analog of L-formed T7 peptide, the d-formed T7 peptide (HRPYIAH), was proposed by Yu et al. (2018) [95]. The authors developed two different formulations of pegylated bilirubin NPs conjugated with D-T7 peptide: one for the delivery of cediranib and the other for paclitaxel. In vitro studies using different cell lines showed that unloaded NPs are biocompatible and that d-T7-modified NPs can penetrate through a BBB model. Conjugation of the NPs with d-T7 peptide significantly improved cell uptake in tumor cells, consequently enhancing the antitumor effect of both formulations. As expected, the concomitant treatment with cediranib-NPs and paclitaxel-NPs led to a better effect than single therapy. After i.v. injection, the data showed that d-T7 modification enhanced the tumor-targeting ability of the NPs, enhancing the antitumor efficacy and prolonging mice survival. Mice bearing intracranial tumors treated with a combination of both formulations showed improved treatment compared with animals treated with a single formulation.

Although to a lower extent, other TfR-targeting peptides have also been explored for drug delivery to GBM tumors, such as T10 (HAIYPRHGGC) and CRTIGPSVC (CRT). These iron-mimicking peptides offer an advantage over conventional T7 and T12 peptides by envisaging the in situ recruitment of the Tf corona on NPs’ surface. This is further discussed in Section 4.5.

### 4.4. Other Strategies

Other less explored strategies have been reported to target the TfR for GBM application such as aptamers. Aptamers are single-stranded oligonucleotides or peptides that fold into defined three-dimensional architectures and bind to molecular targets such as cell receptors. Their popularity has been increasing within the scientific community, because the use of aptamers provides not only the benefits of antibodies such as an equal or even superior affinity/specificity to the target receptor but also offers unique advantages, such as higher stability, smaller sizes, more straightforward modification and immobilization, and higher reproducibility [101].

Fu et al. (2019) developed nucleic acid NPs for temozolomide delivery [102]. Their surface was modified with the GS24 aptamer to improve the brain targeting ability of the NPs. This is a DNA aptamer composed of 64 nucleotides that bind to the TfR. Cytotoxicity studies using a variety of human GBM cells revealed that nanoencapsulation in the developed modified NPs enhanced temozolomide antiproliferative activity, with a more pronounced effect in drug-sensitive cells. Nonetheless, the developed NPs were able to mitigate resistance in drug-resistant cells by decreasing the expression of the repair enzyme O^6^-methylguanine-DNA-methyltransferase (MGMT). This effect was verified because the developed NPs were also functionalized with an extra aptamer (AS1411) that can enhance NPs internalization in the cell nuclei where temozolomide consumes the MGMT enzyme [103]. Another major limitation of CT is the drug resistance mechanisms, with temozolomide’s efficacy being diminished by the activity MGMT [104]. Real-time biodistribution assays in healthy mice proved that the modified NPs could permeate the BBB and accumulate in the brain parenchyma.

### 4.5. Approaches to Overcome Common Challenges of Surface Modification Strategies

Receptor-mediated transcytosis is undoubtedly the most explored mechanism to enhance NPs’ transport across biological barriers such as the BBB and tumor cell membranes. However, the transcytosis efficiency must be carefully considered when designing a nanoformulation. Due to the high affinity of the targeting moieties to the TfR in the BBB cells, ligand-modified NPs are likely to be entrapped within these cells, consequently reducing the number of NPs’ that effectively cross the barrier and reach the GBM cells. To address this issue, strategies based on an acid-cleavable linkage between the ligand and the NPs’ core have been explored for brain targeting [105,106,107].

**Figure 7:**
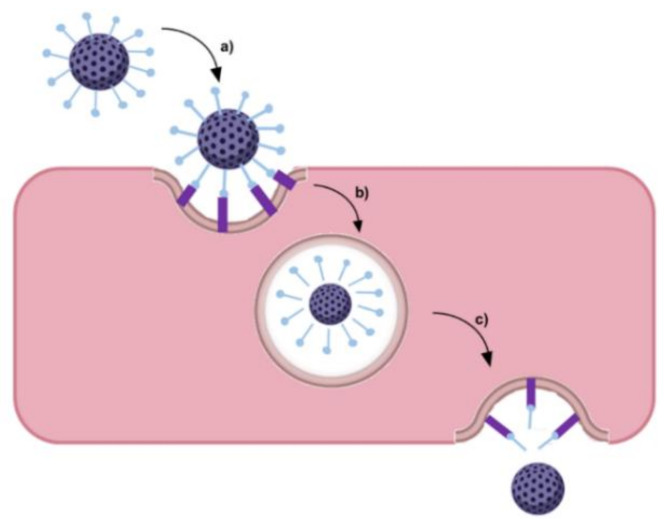
Schematic representation of separation of the NPs from the Tf–TfR complex in the acidic endo/lysosomal compartment: (**a**) ligand-modified NP recognizes and binds to the TfR; (**b**) NP separated from the Tf-TfR complexes in the endosome; and (**c**) NP’s endosomal escape followed by exocytosis.

Ruan et al. (2018) developed poly-l-lysine dendrimers coated with acid-cleavable Tf to deliver doxorubicin [69]. In vitro studies showed that acid-sensitive Tf-modified NPs were efficiently internalized by the BBB cells through a TfR-mediated endocytosis pathway. These studies further revealed that the modification of the NPs with acid-sensitive Tf enhanced the transcytosis efficiency, with the NPs escaping from lysosomes more efficiently than the control NPs modified with acid-non-cleavable Tf. The authors verified that the acid-sensitive Tf was cleaved from the NPs, leading to the separation of the NPs from the Tf–TfR complex due to the acidic environment found in the endo-/lyso-somal compartments as schematized in Figure 7.

However, some authors argue that the cleavage of Tf molecules from the NPs after BBB transcytosis would lead to the loss of NPs’ targeting ability to GBM cells [108]. To prove that NPs retained their targeting efficiency, Ruan et al. (2018) performed further in vitro studies using rat glioma cells to assess the cytotoxicity of the NPs after transcytosis across the BBB model [69]. The obtained results proved that acid-sensitive Tf NPs were more efficient than control NPs in inhibiting glioma cell proliferation, suggesting that these were able to target the glioma TfR-overexpressing cells. Biodistribution studies using mice bearing intracranial tumors showed the higher ability of the acid-sensitive Tf dendrimers to accumulate in the glioma site compared with acid-non-cleavable Tf dendrimers. This enhanced tumor-targeting ability improves the in vivo antiglioma effect while reducing the systemic toxicity in healthy tissues.

**Figure 8:**
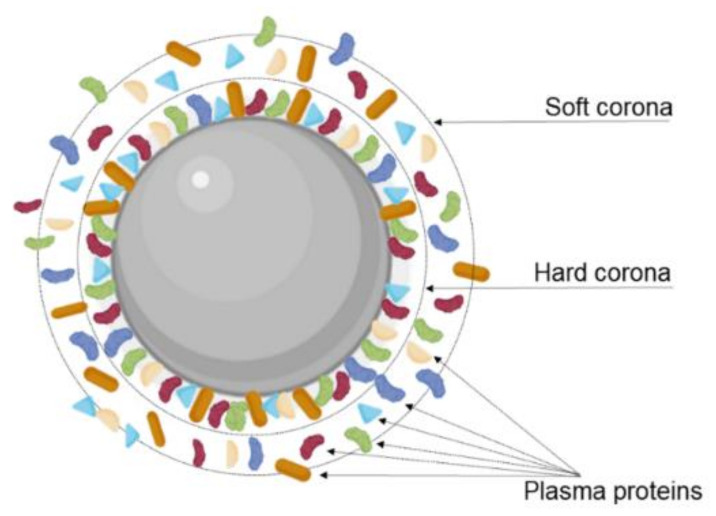
Schematic representation of the protein corona formed in the NPs’ surface. The protein corona is composed of a hard layer, where the proteins are more tightly associated with the NPs’ surface; and a soft layer where the proteins diffuse more freely.

Additionally, the transcytosis efficiency can be affected due to the interactions of the NPs with the biological environment. Modifying the NPs’ surface with targeting ligands, including proteins, peptides, and other molecules, can promote serum protein absorption after i.v. injection and consequently the formation of a protein corona (Figure 8). This protein corona significantly changes the NPs’ properties, impacting their biological performance and posing a major obstacle for targeted delivery [109]. As reported, the protein corona can hamper the interaction between the targeting ligands and the receptor, leading to the loss of the targeting ability. In fact, different authors reported this effect for transferrin-modified NPs after protein corona formation in vitro in the presence of 10% FBS [59] and in vivo [110]. Protein corona can also hinder the lysosomal escape and BBB transcytosis.

Thus, Xiao et al. (2021) studied the influence of protein corona formation on cellular endocytosis, BBB transcytosis, and glioma targeting ability of Tf-modified pegylated polystyrene NPs [64]. For that, the authors prepared NPs coated with protein corona achieved by two pathways. (i) In vitro protein-corona-coated NPs were obtained by incubating the NPs with human plasma proteins, and (ii) in vivo protein corona NPs were obtained by i.v. administration of NPs in mice, followed by blood collection. The authors verified that NPs’ size increased after the protein corona formation both in vitro and in vivo. The surface charge also changed from positive to negative due to the adsorption of negatively charged plasma proteins and partial elimination of Tf-bound molecules on the NPs surface. This work also showed that the protein corona shields the remaining Tf molecules at NPs’ surface, sterically blocking the TfR recognition and affecting BBB transcytosis. In fact, in vitro uptake studies in a BBB monolayer model composed of brain microvascular endothelial cells showed that the Tf-mediated targeting ability of the NPs was lost entirely after in vitro protein corona formation, exhibiting uptake rates similar to the nonmodified control NPs. However, the formation of the protein corona in vivo attenuated these effects being able to preserve part of this targeting ability. The obtained results also showed that the protein corona significantly reduced the lysosomal escape ability of the Tf-modified NPs in the presence of in vivo protein corona while eliminating this ability in the presence of in vitro corona. BBB transcytosis efficiency was also evaluated, and the obtained results depicted a two-fold lower endothelial permeability for NP-corona complexes when compared to Tf-modified NPs, revealing that the formation of the protein corona significantly reduces the BBB transcytosis. The authors also verified that corona proteins disassociate partially from the NPs after BBB transcytosis and that the composition of the protein corona is also qualitatively changed, exhibiting different proteins derived from brain endothelial cells. Interestingly, the authors verified that the NPs retained their Tf-mediated glioma targeting ability after the BBB transcytosis.

The same group also conducted a study to evaluate the impact of ligand type, size, and conformation on protein corona formation [111]. Three different nanoformulations of polystyrene NPs were prepared, one modified with Tf molecules, the other with L-form T7 peptide, and the last with d-T7. The authors concluded that the qualitative and quantitative composition of the protein corona is strongly influenced by NPs’ surface chemistry including ligand type, size, and conformation, which impacts differently the NPs internalization and lysosomal escape [111]. Other physicochemical properties of NPs, such as their size and shape and the biological environment, were also highlighted as major factors regulating protein corona formation [112].

**Figure 9:**
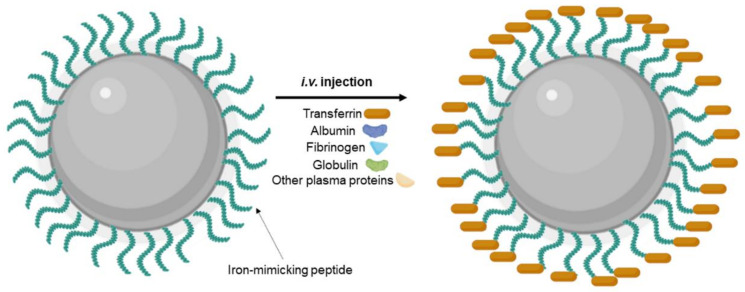
Schematic representation of Tf protein corona formation after i.v. injection of NPs modified with iron-mimicking peptides.

Different strategies were proposed to inhibit nonspecific protein corona adsorption, such as modification with stealth polymers such as PEG, preformed protein corona, and biomimetic membrane camouflage [112]. Precoating with specific proteins/peptides such as iron-mimicking peptides to modulate the formation of protein corona is also an interesting approach. Iron-mimicking peptides such as T10 and CTR promote the in situ recruitment of the Tf corona on NPs [113]. After systemic administration, these peptides bind to the endogenous Tf present in the bloodstream to form a corona around the NPs’ surface, as schematized in Figure 9. This promotes the selective translocation of the NPs across the BBB and their internalization in the target tumor cells. Since these peptides bind to the nonbinding domains of Tf, this nondisruptive binding does not alter the biological function of the endogenous Tf [114]. T10 was employed by Huo et al. (2020) to modify the surface of silica NPs containing doxorubicin [92]. In vitro studies showed that cell uptake in human GBM cells is enhanced by surface modification with T10 and increases with the increase in the density of conjugated peptides. T10 conjugation also inhibited the drug efflux mediated by the P-glycoprotein in GBM cells. Studies with an in vitro BBB model revealed that the Tf corona enhanced NPs transcytosis while preserving BBB integrity. Mice bearing intracranial tumors showed high NPs accumulation in the brain after i.v. injection of the T10-modified NPs. The Tf corona hinders nonspecific adsorption of the other plasma proteins, avoiding NPs clearance by the immune systems, justifying the increased brain targeting ability of the T10-modified NPs comparatively with control NPs. T10-modified NPs exhibited higher tumor growth inhibition efficacy with lower side effects, increasing animal median survival time [92].

CTR peptide which was first identified by Staquicini et al. (2011) and was reported for drug delivery to GBM tumors by Kang et al. (2015) [90]. CRT can selectively bind to a complex of Tf and TfR, inducing allosteric conformational changes that functionally mimic iron molecules, leading to its uptake by the target cells [115]. In this work, the authors developed PLGA NPs to deliver paclitaxel. In vitro studies using rat glioma cells showed that CRT-NPs were more efficiently internalized than control NPs modified with Tf. In vitro studies with 3D spheroid models showed that CRT-modified PLGA NPs have higher penetration ability than Tf-modified NPs. In vitro studies using a BBB model also revealed the higher ability of the CRT-modified NPs to permeate through the barrier. Cytotoxicity studies proved that drug encapsulation in CRT-PLGA NPs enhances the antiproliferation effect of paclitaxel comparatively to drug free or loaded in Tf-PLGA NPs on both glioma cell monolayer and spheroids. Animal studies using mice bearing intracranial tumors proved that surface modification with CRT increased NPs accumulation in the intracranial tumors to a greater extent than Tf-modification, leading to an enhanced antitumor efficacy with prolonged median survival and good biocompatibility in healthy organs [90].

The BBB transcytosis efficiency can also be significantly affected by the ligand density. Therefore, the ligand density should be carefully considered when designing TfR-targeted NPs. Studies have reported that while increasing ligand density provides higher targeting ability, it also may cause quicker blood clearance [116]. In fact, Johnsen et al. (2019) showed that increasing the density of RI7217 antibody in liposomes and gold NPs improved the NPs transport across the mice brain. However, it accelerated NPs’ clearance from the systemic circulation, increasing the off-target accumulation in the spleen. The TfR overexpression in the red pulp and residing macrophages in the spleen can explain the higher accumulation in this organ. Interestingly, increased ligand density also leads to more severe side effects in treated mice [117]. As previously mentioned in this review, significantly increasing the ligand density can also cause the saturation of TfR and consequently decrease BBB transcytosis efficiency. Other authors also observed this effect for NPs modified with Tf molecules [118] or OX26 antibodies [119].

To overcome the limitations of the surface functionalization strategies, alternative approaches were explored, such as producing NPs with a material that has an affinity for the TfR, saving time and costs associated with conjugation methodologies. Ferritin, a protein that contains iron and the primary form of iron stored inside of cells, was proposed to produce NPs for GBM targeting. Liu et al. (2020) developed nanocages of endogenous human ferritin to deliver paclitaxel [120]. In vitro studies using mice brain endothelial cells and rat glioma cells showed that the developed NPs are efficiently taken up by both studied cells. To evaluate if endogenous Tf could inhibit cellular uptake, the TfR was blocked with excess Tf and ferritin before treatment with NPs. The obtained results depicted that while excess ferritin reduces cell uptake, pretreatment with Tf did not significantly affect NPs internalization, proving that endogenous Tf cannot inhibit ferritin binding to the TfR. Furthermore, paclitaxel encapsulation in the developed NPs proved to increase by 20-fold the drug permeability through a BBB in vitro model of mice brain endothelial cells. Nanoencapsulation in ferritin nanocages also enhanced drug penetration ability in 3D spheroid models, leading to enhanced antiproliferative activity. Real-time and postmortem biodistribution studies using mice bearing intracranial tumors proved that nanoencapsulation increased by 10-fold drug accumulation in the brain tissue after i.v. administration, while decreasing drug accumulation in the liver. The improved bioavailability of the drug led to increased mice survival with no toxicity to healthy organs.

## 5. Critical Opinion and Future Perspectives

The BBB is a dynamic structure that blocks the passage of most drugs into the brain, limiting the efficacy of the therapy of neurological diseases such as GBM. Hence, the scientific community has concentrated efforts on finding suitable solutions to overcome this barrier and increase drug accumulation in brain tumors. Different strategies have been proposed, from more invasive strategies such as intracerebral injection to less invasive strategies such as induced BBB disruption. BBB can be transiently disrupted by the administration of chemical agents due to hyperosmolarity or by applying FUS [121]. However, this can damage the BBB, limiting the implementation of this approach for GBM treatment. Other severe toxic effects can be observed such as rebound intracranial hypertension, electrolyte disturbance, and kidney failure [122].

DDS have been proposed as a safer strategy to enhance drug delivery to the brain. However, the capacity of DDS to cross the BBB depends greatly on their physicochemical features. For example, lipid NPs can effectively permeate through this biological barrier, but their lipophilicity can also enhance drug accumulation in other organs and cause deleterious side effects. Therefore, it is of utmost importance to employ active targeting strategies to direct these carriers to the target tissue. Different authors proposed the use of positively charged molecules to modify the surface of NPs to increase their transport across the BBB [123]. However, cationic NPs are usually associated with higher toxicity than neutral or negatively charged NPs.

**Figure 10:**
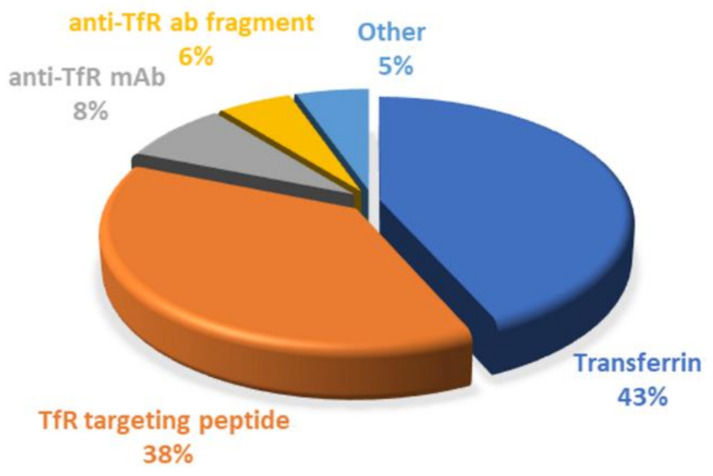
Graphical representation of the distribution of the TfR-targeting started for GBM therapy. This chart was created based on the works reported in this review.

Since it has been demonstrated that BBB cells express several receptors on their surface, targeting strategies based on receptor-mediated transcytosis have been highlighted as a suitable solution. TfR is very concentrated in brain tumors, compared to other tissues, making it a desirable target for enhanced drug delivery for GBM treatment, and this review demonstrated that different approaches are being explored to target the TfR, as shown in Figure 10.

Surface conjugation with Tf molecules is undoubtedly the most explored strategy so far, corresponding to around 43% of the employed strategies. The main advantages of using Tf as a ligand are mainly related to being easily obtained from human sources, at high abundance and at relatively low cost. Furthermore, human Tf is nonimmunogenic; therefore, it can be safely administered without causing toxicity [55].

However, Tf exhibits some limitations such as the loss of specificity in the biological environment due to the high levels of endogenous Tf. Plasmatic Tf molecules can decrease the affinity of Tf-conjugated NPs for the TfR by competitive binding and therefore leading to decreased therapeutic efficacy. To overcome these drawbacks, other strategies have been explored through the use of ligands that bind noncompetitively to an alternative site of the receptor [83], such as antibodies against the TfR. These alternative ligands do not compete with the Tf found in blood circulation, avoiding the saturation of the TfR and increasing specificity [124].

Therefore, monoclonal antibodies have been widely explored for brain delivery. For GBM therapy, only the RVS10 and OX26 clones were reported, but different clones against the TfR have been investigated by the scientific community, such as RI7217 and 8D3 clones, which also have the potential to be envisaged for GBM application. For example, Salvati et al. (2013) used RI7217 monoclonal antibodies to functionalize liposomes for the transport across the BBB and target the amyloid-β peptide. The modification with RI7217 monoclonal antibodies proved to increase the permeability of the nanocarriers through the BBB in vitro model [74]. Cabezón and colleagues (2015) modified the surface of gold NPs using the 8D3 anti-TfR monoclonal antibody. Animal studies using mice showed the that BBB cells efficiently internalized the nanocarriers [75].

However, the synthesis of monoclonal antibodies, as to control their quality, is difficult, limiting their clinic application. In addition, the high molecular weight of antibodies and Tf molecules poses an obstacle for their use, motivating the need to find more effective targeting strategies. In recent years, scFvs against the TfR have been explored as an alternative to full-length antibodies. scFvs fragments preserve the binding specificity of the antibodies but exhibit better pharmacokinetic properties due to having a higher ability to penetrate the tissues and faster blood/tissue clearance due to their smaller dimensions [125]. Despite all the advantages of using antibodies and their fragments, their clinical application would considerably increase treatment costs. Thus, antibody-based strategies, including scFv, correspond only to 14% of the works reported in this review.

As a result, the search for more cost-effective alternatives has increased. TfR-targeting peptides have recently gained popularity due to their benefits, representing 38% of the explored strategies. Similar to antibodies, these also bind to different sites of the TfR, not competing with the plasmatic Tf. In addition, the smaller dimensions of these peptides represent a major advantage over the use of antibodies and Tf molecules. Studies have reported that ligands with high molecular weight can prevent or reduce the modified-NPs’ transport across the BBB. Thus, surface modification with small TfR targeting peptides can maintain binding efficiency, high receptor specificity, and low metabolic consequences [96].

However, high clearance and poor pharmacokinetics are common drawbacks of targeting peptides since proteases in blood circulation can induce their proteolysis [126]. The biological performance of a targeting peptide directly depends on its serum stability. Thus, peptides with improved stability have been proposed [127]. Retroinverso peptides, d-peptides synthesized from d-amino acids, retain the topological order of the parental l-peptides due to the inversion of the peptide bonds but present the advantage of increasing enzymatic stability and the plasma half-life. In particular, l-T7 peptide is widely explored for other applications different from GBM, and it was reported to have higher receptor affinity in comparison with its l-parent peptide [128]. Retro-d-peptides for T12 have also been described [126,129,130,131], but to the extent of our knowledge, these were not yet envisaged for GBM targeting.

Alternatively, strategies that do not resort to surface conjugation have gained popularity to save time and associated costs. For example, ferritin self-assembled nanocages have been described as promising carries. This protein is present in the human body and plays an important role in iron storage, therefore not exhibiting immunogenicity. Ferritin is a strong endogenous binding ligand for TfR with advantages for GBM therapy because it is not competitively inhibited by endogenous Tf. Ferritin is a spherical nanocage composed of two chains, a heavy (H) and light (L) chain, and a central space that can be used to carry drugs. The H chain is the one that specifically binds to the TfR and exhibits binding epitopes different from Tf, resulting in a noncompetitive binding even with a high concentration of endogenous Tf [120].

Despite all efforts and decades of development, minimal clinical progression for GBM therapy has been made. Less than 10 trials of DDS for GBM appear registered in ClinicalTrials.gov (either recruiting/ongoing/completed), with only one nanoformulation targeting the TfR. A phase II clinical trial sponsored by SynerGene Therapeutics is studying the effect of the combined therapy of free temozolomide with liposomes modified with anti-TfR scFv for the delivery of the p53 tumor-suppressor gene in GBM patients (ClinicalTrials.gov Identifier: NCT02340156). p53 can inhibit the activity of the DNA-repair protein MGMT. MGMT plays a major role in therapy failure since it can repair drug-induced DNA damage, reverting therapeutic effects [132]. In another clinical trial, this DDS has previously shown promising results for the treatment of solid tumors [133].

Other TfR-based strategy was studied in clinical trials for GBM therapy but without using NPs as DDS. In this study, the authors conjugate Tf molecules to the diphtheria toxin protein (Tf-CRM107) to enhance its brain uptake, and this showed promising results with tumor volume reduction without systemic toxicity in a phase II clinical trial with 44 GBM patients [134]. A further phase III clinical trial was initiated, but it was withdrawn due to the assumption that Tf-CRM107 would not meet FDA criteria for efficacy (ClinicalTrials.gov Identifier: NCT00083447).

Further advances in this field are required to expand the number of TfR-targeting DDS in clinical trials to enhance the therapeutic potential of strategies targeting this receptor for GBM therapy. Additionally, there are a few clinical trials for the evaluation of TfR-targeting DDS for the treatment of other tumors, such as solid tumors (ClinicalTrials.gov Identifiers: NCT00689065, NCT00355888) that could have the potential to be applied for GBM treatment. Moreover, the translational aspect of interspecies differences in TfR expression and function should be considered for the successful advance of TfR-targeted DDS for clinical application.

Several factors contributed to the nonexistence of clinically available DDS for the treatment of GBM; however, it is still crucial to go further in the research field to generate more efficient DDSs to ultimately treat this devastating disease. Strategies to overcome resistance mechanisms associated with GBM should be prioritized in DDS research. Since therapy failure is not only associated with low bioavailability in the tumor tissue and toxicity in the healthy organs but also closely associated with intrinsic resistance mechanisms, it is of paramount importance to address these issues—in particular, the resistance mechanisms mediated by the MGMT protein. The methylation status of MGMT is a strong prognostic factor in GBM patients and 40–60% of GBM patients show unmethylated MGMT (not-silenced gene) [132]. Therefore, strategies to overcome this can significantly improve therapeutic outcomes.

Additionally, most of the research reported in this review focused on drug delivery with less than 20% of the studies aiming to develop new imaging and diagnosis tools. As efficient early diagnosis can provide better therapeutic outcomes, developing effective diagnosis tools is crucial.

## 6. Conclusions

GBM is an aggressive brain tumor that is still incurable, and finding novel effective therapeutic solutions is urgent within the scientific community. During the last decades, NPs have emerged as promising tools to overcome the drawbacks of chemotherapy, such as low bioavailability in the target cells and high toxicity to the healthy tissues. Though, passive targeting strategies that rely solely on the physicochemical features of NPs most times revealed to be ineffective due to lacking tissue specificity. Hence, brain tissue being upregulated with TfR led to the concept of TfR targeted anticancer therapeutics. Thus, currently, active targeting strategies focusing on this receptor remain undoubtedly the most popular approach to enhance drug delivery for the treatment of GBM and other neurological diseases. Despite the scientific advancements in recent years, little progress was verified in clinical trials. High costs and complex production methodologies are generally associated with a low success rate.

Further insightful works are necessary to optimize TfR targeting to boost the number of effective and reliable DDS in clinical trials. The great potential of TfR-targeting NPs for drug delivery in GBM must be deeply explored to introduce in the market a nanotech product with potential for clinical application able to overcome the limitations of the current therapeutic approaches. This will greatly impact cancer research and ultimately will contribute to saving GBM patients’ lives.

## Figures and Tables

**Table 1:** Cellular markers used in GBM therapies.

Cell Markers	Type	Refs.
A2B5	Surface glycoside	[38]
CD15	Cell surface protein	[39]
CD44	Cell surface marker	[40]
CD133	Surface glycoprotein	[41]
EGFR	Transmembrane protein	[42]
VEGF	Signal protein	[41]
IDH1	Transcriptional regulator	[43]
IL-13	Surface receptor	[44]
Integrin α5β3	Adhesion molecule	[45]
Integrin α6	Transmembrane receptor	[46]
L1CAM	Adhesion molecule	[47]
MMP-2	Matrix metalloproteinase	[48]
TfR	Transmembrane glycoprotein	[49]

**Table 2:** Currently developed transferrin-modified nanosystems for GBM therapy.

Nanocarrier	Coating	Loaded Content	Size (nm)	Surface Charge	Development Phase		Refs.
					Cellular Studies	Animal Studies	
Liposomes	PEG	Cisplatin	294	Positive	C6: cytotoxicity; bEnd3: permeation studies on BBB model	n.a.	[56]
		Zoledronic acid	147	Positive	U373: cytotoxicity studies	Male nude mice bearing intramuscular or orthotopic xenografts: biodistribution and tumor growth inhibition studies	[57]
		Magnetic iron oxide NPs and quantum dots	179	Negative	U87: cytotoxicity and uptake studies	n.a.	[58]
		Resveratrol	211	Negative	U87: cytotoxicity and uptake studies	Female nude mice bearing subcutaneous tumor xenografts: biodistribution and tumor growth inhibition studies	[59]
	TPGS	Docetaxel and quantum dots	183	Neutral	n.a.	Charles Foster rats: biodistribution studies	[60]
PLA NPs	PEG	Doxorubicin	100	Negative	C6: cytotoxicity and uptake studies	Rat bearing intracranial tumor xenograft: biodistribution and tumor growth inhibition studies	[61]
		n.a.	95–110	Negative	C6: uptake studies	Male rats bearing orthotopic intracranial tumor: biodistribution studies	[62]
Chitosan NPs	PEG	Docetaxel	285	Negative	C6: cytotoxicity and uptake studies	Male/female rats: pharmacokinetic studies	[63]
Polystyrene NPs	PEG	n.a.	84	Positive	C6: uptake studies; bEnd3: transcytosis studies on BBB model	Male mice: i.v. administration of NPs to form protein corona	[64]
Silicon NPs	None	n.a.	182	Negative	U87: cytotoxicity, transfection, migration and uptake studies	n.a.	[65]
Silicon NPs	None	Doxorubicin	167	Negative	U87: cytotoxicity and uptake studies; hCMEC/D3: permeation studies on BBB model	n.a.	[66]
Indocyanine green NPs	None	ICG	12	Negative	U87: cytotoxicity and uptake studies; U87/bEnd3: permeation studies on BBB model	Nude mice bearing subcutaneous tumor and intracranial tumor: bioimaging and biodistribution studies; tumor growth inhibition and safety studies	[67]
PAMAM dendrimers	PEG	Temozolomide	w/o info	w/o info	Patient-derived cells: cytotoxicity and uptake studies	Male nude mice bearing intracranial tumor: biodistribution and tumor growth inhibition studies	[68]
Poly-l-lysine dendrimers	MAN	Doxorubicin	29	Positive	C6: uptake and apoptosis studies; bEnd3: permeation studies on BBB model	Male nude mice bearing intracranial tumor: biodistribution and tumor growth inhibition studies	[69]
Ruthenium NPs	none	[Ru(bpy)_2_(tip)]^2+^	125	Positive	U87: cytotoxicity and uptake studies; HBMEC: permeation studies on BBB model	Male nude mice bearing intracranial tumor: biodistribution and tumor growth inhibition and safety studies	[70]
Iron oxide NPs	PEG	siRNA against the polo-like kinase I (siPLK1)	60	Positive	U87: cytotoxicity and uptake studies; bEnd3: permeation studies on BBB model	Mice bearing intracranial tumor: biodistribution and tumor growth inhibition studies	[71]

**Table 3:** Currently developed antibody-tailored nanosystems for GBM therapy (* mAb–monoclonal antibodies).

Nanocarrier	Ligand	Coating	Drug	Size (nm)	Surface Charge	Development Phase		Refs.
						Cellular Studies	Animal Studies	
Liposomes	OX26 mAb	PEG	Cisplatin	157	Negative	C6: uptake studies BCECs: permeation studies on BBB model	Wistar rats bearing intracranial tumor: biodistribution studies, safety of NPs, and animal survival	[78]
	scFv against the TfR	None	Temozolomide	40	Positive	U251 and U87: cytotoxicity, transfection, and uptake studies	Female athymic mice bearing intracranial tumor: biodistribution, tumor growth inhibition, and safety studies	[79]
	scFv against TfR	None	p53 tumor-suppressor gene	114	Positive	U87, U251, T98G and LN-18: cytotoxicity studies	Female athymic nude mice bearing intracranial tumors: tumor growth and biodistribution studies	[80]
PLGA NPs	OX26 mAb	PEG	Temozolomide	194	Negative	U251 and U87: cytotoxicity and uptake studies; HBLECs: permeation studies on BBB model	n.a	[81]
Poly(β-l-malic acid) NPs	RVS10 mAb	PEG	Temozolomide	15	Negative	U87 and T98G: cytotoxicity and uptake studies	n.a.	[82]

**Table 4:** Currently developed nanosystems modified with TfR-targeting peptide for GBM therapy.

Nanocarrier	TfR-Targeting Peptide	Coating	Loaded Content	Size (nm)	Surface Charge	Development Phase		Refs.
						Cellular Studies	Animal Studies	
Liposomes	T7	PEG	siRNA	83	Positive	U87: transfection and uptake studies; BMVECs: permeation studies on BBB model	Male nude mice bearing intracranial tumor: biodistribution, tumor growth inhibition, and safety studies	[84]
	T12	PEG	Vinblastine	100	Negative	GBM cells and stem cells: cytotoxicity and uptake studies; permeation studies on BBB model	Male nude mice bearing intracranial tumor: biodistribution and tumor growth inhibition studies	[85]
SLNs	T7	Blood cell membrane	Vincristine	124	Negative	C6: cell binding and cytotoxicity studies; HUVEC and bEnd.3: permeation studies on BBB model	Male/female IRC mice bearing intracranial xenografts: tumor growth inhibition, biodistribution, and safety studies	[86]
Nanocomplexes of myristic acid	T12	none	siRNA	85–100	Positive	U87: cytotoxicity, uptake and transfection studies; b.End3: permeation studies on BBB model	n.a.	[87]
PLGA NPs	T7	PEG	Seliciclib	127	Negative	U87: cytotoxicity and uptake studies	n.a.	[88]
	T7	PEG	Iron oxide NPs, paclitaxel and curcumin	130	Negative	U87: cytotoxicity and uptake studies; bEnd.3 cells: permeation studies on BBB model	Male nude mice bearing intracranial xenografts: tumor growth inhibition study	[89]
	CRTIGPSVC	PEG	Paclitaxel	118	Negative	C6: cytotoxicity and uptake studies; BCEC: permeation studies on BBB model	Nude mice bearing intracranial tumor: biodistribution and tumor growth inhibition studies	[90]
PLA micelles	T12	PEG	Paclitaxel	110	Negative	U87 U118: cytotoxicity and uptake studies; HUVEC: permeation studies on BBB model	Male nude mice bearing subcutaneous glioma: tumor growth inhibition studies	[91]
Silica NPs	T10	PEG	Doxorubicin	168	Positive	U87 and C6: cytotoxicity and uptake studies; bEnd.3: permeation studies on BBB model	Male nude mice bearing intracranial tumor: biodistribution, tumor growth inhibition, and safety studies	[92]
Poly-l-lysine dendrimers	T7	None	Doxorubicin and pORF-hTRAIL gene	173	Positive	U87: cytotoxicity, uptake and transfection studies	Male nude mice bearing intracranial tumor: biodistribution and tumor growth inhibition studies	[93]
	T7	PEG	siRNA	141	Positive	U87: cytotoxicity, uptake and transfection studies; BCECs: permeation studies on BBB model	Male nude mice bearing intracranial tumor: biodistribution and tumor growth inhibition studies	[94]
Bilirubin NPs	D-T7	PEG	Cediranib or Paclitaxel	112–118	Positive	HUVE, C6 and bEnd: cytotoxicity; C6 and bEnd.3: uptake studies; bEnd.3: permeation studies on BBB model	Male mice bearing intracranial tumor: pharmakokinetics, biodistribution, safety, and tumor growth inhibition studies	[95]
Gold NPs	T7	PEG	Phthalocyanine 4	41	Negative	LN229 and U87: cytotoxicity and uptake studies	Mice bearing intracranial tumor: biodistribution studies	[96]
Exosomes	T7	None	Antisense miRNA oligonucleotides	15–50	Negative	C6: cytotoxicity and uptake studies	Male rats bearing intracranial tumor: biodistribution and tumor growth inhibition studies	[97]
